# Relationships amongst osteoarthritis biomarkers, dynamic knee joint load, and exercise: results from a randomized controlled pilot study

**DOI:** 10.1186/1471-2474-14-115

**Published:** 2013-03-27

**Authors:** Michael A Hunt, Courtney L Pollock, Virginia Byers Kraus, Tore Saxne, Sue Peters, Janet L Huebner, Eric C Sayre, Jolanda Cibere

**Affiliations:** 1 Department of Physical Therapy, University of British Columbia, BC, Vancouver, Canada; 2 Arthritis Research Centre of Canada, BC, Vancouver, Canada; 3 Duke School of Medicine, NC, Durham, USA; 4 Department of Clinical Sciences, Section of Rheumatology, Lund University, Lund, Sweden; 5 Department of Medicine, University of British Columbia, BC, Vancouver, Canada

## Abstract

**Background:**

Little is known about the relationships of circulating levels of biomarkers of cartilage degradation with biomechanical outcomes relevant to knee osteoarthritis (OA) or biomarker changes following non-pharmacological interventions. The objectives of this exploratory, pilot study were to: 1) examine relationships between biomarkers of articular cartilage degradation and synthesis with measures of knee joint load during walking, and 2) examine changes in these biomarkers following 10 weeks of strengthening exercises.

**Methods:**

Seventeen (8 male, 9 female; 66.1 +/- 11.3 years of age) individuals with radiographically-confirmed medial tibiofemoral OA participated. All participants underwent a baseline testing session where serum and urine samples were collected, followed by a three-dimensional motion analysis. Motion analysis was used to calculate the external knee adduction moment (KAM) peak value and impulse. Following baseline testing, participants were randomized to either 10 weeks of: 1) physiotherapist-supervised lower limb muscle strengthening exercises, or 2) no exercises (control). Identical follow-up testing was conducted 11 weeks after baseline. Biomarkers included: urinary C-telopeptide of type II collagen (uCTX-II) and type II collagen cleavage neoepitope (uC2C), serum cartilage oligomeric matrix protein (sCOMP), serum hyaluronic acid (sHA) and serum C-propeptide of type II procollagen (sCPII). Linear regression analysis was used to examine relationships between measures of the KAM and biomarker concentrations as baseline, as well as between-group differences following the intervention.

**Results:**

KAM impulse predicted significant variation in uCTX-II levels at baseline (p = 0.04), though not when controlling for disease severity and walking speed (p = 0.33). KAM impulse explained significant variation in the ratio uCTX-II;sCPII even when controlling for additional variables (p = 0.04). Following the intervention, changes in sCOMP were significantly greater in the exercise group compared to controls (p = 0.04). On average those in the control group experienced a slight increase in sCOMP and uCTX-II, while those in the exercise group experienced a reduction. No other significant findings were observed.

**Conclusions:**

This research provides initial evidence of a potential relationship between uCTX-II and knee joint load measures in patients with medial tibiofemoral knee OA. However, this relationship became non-significant after controlling for disease severity and walking speed, suggesting further research is necessary. It also appears that sCOMP is amenable to change following a strengthening intervention, suggesting a potential beneficial role of exercise on cartilage structure.

**Trial registration:**

Clinicaltrials.gov NCT01241812

## Background

Osteoarthritis (OA) is a common chronic health condition resulting in significant personal and economic burdens. In the absence of a cure, the main areas of research to-date have centred on developing treatments that can improve symptoms of pain and physical dysfunction as well as improving methods to diagnose the disease and to monitor progression. Most commonly affecting the knee, much of the current literature in these two research areas has been conducted in populations with knee OA.

Plain radiographs remain the most commonly used clinical method of assessing joint structure for the purposes of OA diagnosis and classification of severity. Magnetic resonance imaging (MRI) has also been used to assess changes in the morphology of the bone and cartilage to supplement radiographical findings [1]. Though these approaches are reliable and well-established clinically, their use for the monitoring of changes in cartilage over time is limited by the fact that these changes take place over long periods of time and simply detail soft tissue damage that has already occurred [2]. Indeed, a recent systematic review showed greater responsiveness of radiographic joint space width measurements in studies using follow-up periods of greater than 2 years [3]. Given that significant changes in symptoms and joint structure can occur over the course of 2 years, coupled with the fact that most non-surgical and non-pharmacological interventions are conducted over months instead of years, improved methods of assessing changes in cartilage structure and outcomes following shorter-terms treatments over shorter periods of time are needed.

Joint tissue-related biomarkers in the blood and urine have been used to further the understanding of the pathogenesis of knee OA. Many biomarkers are produced during the synthesis or degradation of articular cartilage and are found in different concentrations based on the presence and severity of knee OA [4,5]. For example, the biomarkers C-propeptide of type II procollagen (CPII), hyaluronic acid (HA), and cartilage oligomeric matrix protein (COMP) in serum, as well as C-telopeptide of type II collagen (CTX-II) and type II collagen cleavage neopeptide (C2C) in the urine have all been shown to be elevated based on the presence and severity of knee OA [6]. Importantly, given that their concentrations reflect processes directly implicated in the synthesis or degradation of articular cartilage, analysis of biomarker concentrations may represent an effective method of assessing cartilage structure over shorter periods of time than conventional methods such as radiography or MRI. However, much is still unknown about these biomarkers, including factors involved in their production and responses to non-pharmacological interventions.

Excessive joint loading is a recognized risk factor for breakdown of articular cartilage based on early *in vitro* studies [7]. These findings have been supported by later gait analysis studies showing a significantly higher rate of knee OA progression over six years in people with high baseline peak external knee adduction moment (KAM) values [8] – a valid and reliable measure of medial compartment knee joint load during walking [9-11] – as well as a relationship between knee cartilage volume loss over twelve months and the baseline KAM impulse [12] – the time integral of the KAM during stance [13]. A link between joint load and biomarker concentrations has also been reported. Piscoya et al. [14] have shown increases in COMP production in response to dynamic mechanical load in cartilage explants, and acute bouts of moderately intense physical activity have been shown to temporarily increase the concentration of sCOMP in healthy individuals [15] as well as those with knee OA [16,17]. However, the relationships between other cartilage biomarkers and measures of everyday joint loading, such as the KAM, are less well known. Further, the effects of common non-pharmacological interventions on biomarker concentrations are also not known.

Given the paucity of research on the relationships of OA biomarkers with joint loading and changes following exercise, the purpose of the present exploratory, pilot study was to address these two knowledge gaps in a sample of individuals with knee OA. Specifically, the primary objective of this study was to determine the relationships between different biomarker concentrations and knee joint load, as measured by the KAM. The secondary objective was to examine changes in biomarker concentrations following a 10-week muscle strengthening intervention.

## Methods

### Participants

Community-based volunteers over the age of 50 years were recruited through advertisements in local newspapers. All had OA in at least one knee according to the American College of Rheumatology classification criteria [18] and reported average knee pain >3/10 on most days of the previous month. All participants had varus alignment and OA predominantly in the medial tibiofemoral compartment. Exclusion criteria included: history of knee replacement surgery or high tibial osteotomy; any knee surgery or corticosteroid injections within the previous 6 months; currently participating in, or intention to begin structured lower limb strengthening exercises within the next 3 months; inability to complete exercises at home or attend 5 visits with the study physiotherapist; and, BMI > 35 to reduce soft tissue artifact of marker movement during quantitative gait analysis. For this pilot study, our aim was to recruit between 15 and 20 individuals. This study was approved by the Clinical Research Ethics Board of the University of British Columbia and all participants provided written informed consent prior to enrollment.

### Procedure

**Figure 1:**
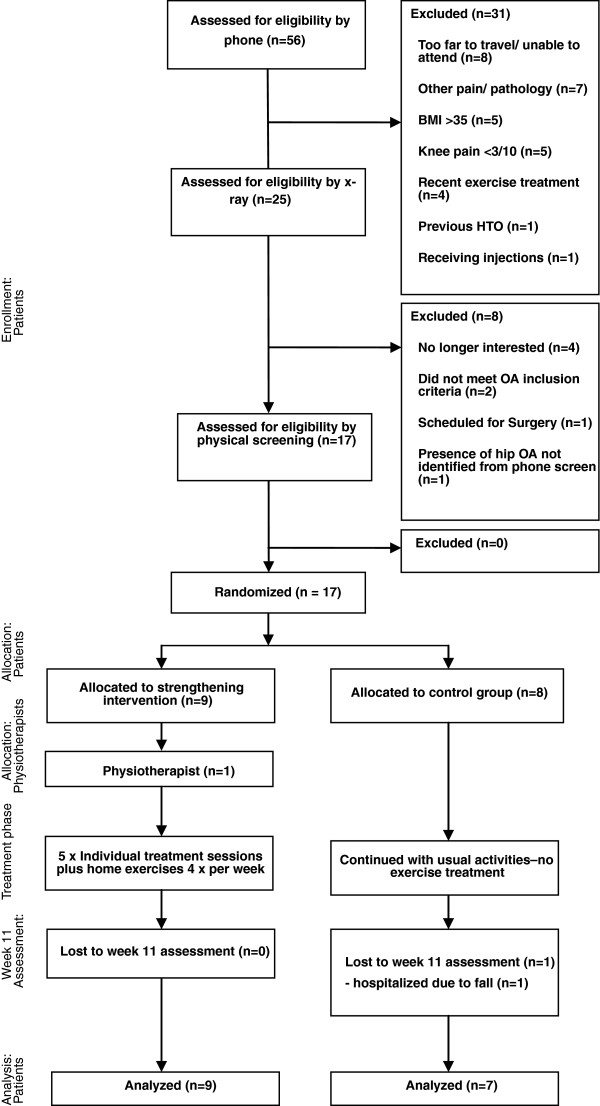
Study participant flowchart.

A pilot 10-week, randomized, assessor-blinded, controlled trial was conducted (Figure 1). Interested participants underwent an initial phone screening based on the above-mentioned inclusion and exclusion criteria. Next, standing posteroanterior radiographs were obtained and assessed for radiographic severity using the Kellgren and Lawrence (KL) classification system [19], and measured for lower limb alignment using published regression equations for short-film radiographs [20]. Those meeting all inclusion criteria were invited to attend a baseline testing session in our laboratory. In cases of bilateral symptoms and radiographic degeneration, the knee reported to be the most painful was determined to be the study limb. Upon completion of data collection, participants were randomly allocated to either the exercise or no exercise (control) group. Randomization was conducted by an investigator not involved with outcome assessment using sealed and consecutively numbered opaque envelopes containing the group allocation information according to a computer-generated random number.

### Interventions

Those randomized to the exercise group received a set of 6 exercises designed to strengthen the hip abductors (standing and side lying hip abduction), quadriceps (standing lunges, mini-squats, seated knee extension), and hamstrings (flexion to 90 degrees while maintaining standing balance on the contralateral limb – with hand support if required) muscle groups. When applicable, additional resistance was provided using ankle cuff weights to achieve sufficient exercise intensity for participants to complete 3 sets of 10 repetitions for each exercise (defined as the largest weight that the participant could successfully and safely use to complete the 3 sets). Exercises were performed a minimum of 4 days per week at home for 10 weeks, and exercise performance and safe progression of resistance was monitored across five visits with the study physiotherapist (MAH) at weeks 1, 2, 3, 5, and 8 of the intervention. Adherence was quantified as the number of home exercise days completed (maximum of 40 days) as well as the number of physiotherapy visits (maximum of 5 visits), converted to a percentage.

Those in the control group did not receive any additional intervention for the 10-week duration of the study and were instructed to maintain their usual clinical management for knee OA.

### Outcome measures

Outcome assessment was conducted at baseline and at follow-up (11 weeks) by an assessor blinded to group allocation. Each assessment session included the collection of blood and urine, three dimensional gait analysis, and strength assessment. All outcome measurements were completed within a 2-hour period at the same facility.

1) Biomarker assessments

Serum and urine samples were collected following a 30 minute rest period in which the participant remained seated. All samples were processed and immediately stored at –20°C, then transferred within one week to a –80°C freezer until analysis. Analyses were conducted at two sites by investigators with previous biomarkers analysis experience [4]. Serum concentrations of CPII (IBEX, Montreal, Canada) were analyzed using an enzyme-linked immunosorbent assay (ELISA) designed to detect the carboxy-propeptide that is released from type II collagen following new procollagen synthesis. Serum HA (Corgenix, Broomfield, USA) was analyzed by a sandwich ELISA utilizing HA binding protein as the capture molecule. Serum COMP was analyzed using a sandwich ELISA that uses antibodies directed against known antigenic determinants of human COMP (AnaMar, Lund, Sweden). CTX-II (IDS, Bolton, UK) in the urine was quantified using an ELISA based on a sequence found exclusively in human type II collagen. Urinary C2C was measured using an ELISA (IBEX, Montreal, Canada) that measures the carboxy-terminus of the primary type II collagen cleavage generated by collagenases. Urinary CTX-II and C2C were corrected for creatinine excretion levels quantified by ELISA (Quidel, San Diego, USA). Intra-assay coefficients of variation were as follows: sCPII, 4.7%; sHA, 5.4%; sCOMP 2.8%; uCTXII, 2.8%; uC2C, 3.5%. All samples yielded measurable concentrations for all 5 biomarkers. All analyses were conducted in duplicate and blinded to treatment status.

2) Knee joint loading during walking

Participants underwent three-dimensional gait analysis while walking barefoot and at a self-selected speed. Reflective markers were positioned over lower limb anatomical landmarks during walking as well as over the medial femoral epicondyles and medial malleoli during an initial static standing trial used to determine joint centre locations. Kinematic data were collected using 8 high-speed digital video cameras (Motion Analysis Corp., Santa Rosa, CA) sampling at 120 Hz. Kinetic data were sampled at 1200 Hz using 2 floor-mounted force platforms (Advanced Mechanical Technology Inc., Watertown, MA) positioned in the middle of the walkway and synchronized with the cameras. Net joint moments were calculated using commercially available software (Orthotrak, Motion Analysis Corp., Santa Rosa, CA). The peak KAM (maximum value during stance) was identified, and the KAM impulse (positive area under the KAM-time curve) was calculated, for each trial and averaged across a total of 5 trials with clean force platform strikes by the study limb.

3) Other measures

Isometric muscle strength was assessed using dynamometry. Isometric knee extension and flexion strength was measured using an isokinetic dynamometer (Biodex, Shirley, NY) while the participant was seated and the knee placed in 40 degrees of flexion. Isometric hip abduction strength was measured using a handheld dynamometer (Hoggan Health, West Jordan, UT) placed over the lateral femoral epicondyle. For each muscle group, participants performed three repetitions of maximal voluntary isometric contractions for five seconds each. The maximum force production across the three trials was averaged and converted to a torque by multiplying by the lever arm, then normalized to body mass (Nm/kg). Participants randomized to the exercise groups completed a weekly diary to record adherence to the home exercise program.

### Statistical analysis

Biomarker data were log-transformed and used as dependent variables in linear regression models with joint load (either peak KAM or KAM impulse) used as the independent variable to predict variance in each biomarker, while controlling for age and sex [21], with additional models also controlling for KL grade and walking speed. Group membership (exercise or no exercise) was added in regression models to predict change in log biomarker levels while adjusting for age and sex, using intention-to-treat principles. Finally, changes in KAM peak and impulse as well as muscle strength were analyzed using repeated measures analysis of variance for descriptive purposes as an indication of the biomechanical and functional effects of the intervention.

## Results

Seventeen participants (8 males, 9 females; mean (SD) age = 66.1 (11.3) years, BMI = 27.0 (4.5) kg/m^2^) were enrolled in the study. Ten participants had mild OA (KL 2), five had moderate OA (KL 3), and two had severe OA (KL 4). Baseline demographic and clinical data were similar between the two groups. Sixteen participants (five males and four females from the exercise group and three males and five females from the control group) returned for the follow-up assessment a mean (SD) of 76.3 (5.3) days after the baseline assessment. Blinding of group allocation to the assessor was maintained for all participants. The time of sample collection was consistent between baseline and follow-up for each participant with the mean (SD) difference being 20 (21) minutes (maximum = 65 minutes). Most (13/17) participants provided samples and underwent biomechanical testing in the morning prior to 11:00 am. Attendance at the supervised physiotherapy sessions was high in the exercise group with participants attending a mean (SD) of 91% (14%) of the sessions. Home exercise adherence rates were also high with a mean (SD) of 83% (17%) of the exercise days completed.

Across all participants at baseline, and while adjusting for age and sex, KAM impulse predicted significant variation in uCTX-II levels (β = 1.19, 95% CI = 0.16, 2.21; *p* = 0.05) as well as the uCTX-II:sCPII ratio (β = 1.50, 95% CI = 0.72, 2.28; *p* < 0.01). However, when KL grade and walking speed were added to the regression models, the relationship with uCTX-II became non-significant (β = 0.58, 95% CI = -0.53, 1.68, *p* = 0.33), while the significant relationship with uCTX-II:sCPII remained (β = 1.11, 95% CI = 0.15, 2.07; *p* = 0.04). In contrast, peak KAM was not able to explain any significant amount of variation in any biomarker or ratio when accounting for age and sex or when adding KL grade and walking speed to the models (*p* > 0.34). No other significant findings were found for either measure of KAM in any biomarker regression model.

When comparing changes between groups following the intervention (Table 1), significantly greater reductions in sCOMP (β = 0.16, 95% CI = 0.02, 0.30; *p* = 0.04) as well as slightly greater, non-significant reductions in uCTX-II (β = 0.33, 95% CI = 0.04, 0.71; *p* = 0.11) were observed in the exercise group compared to those in the control group. No other significant between-group differences existed in any single biomarker outcome or biomarker ratio. Finally, no significant between-group differences were observed in any gait or strength outcome (*p* > 0.11).

**Table 1:** Mean (SD) values for KAM and strength outcomes as well as for log-transformed, unadjusted biomarker levels and ratios of degradation to synthesis (sCPII) at baseline and follow-up for each group

	**Baseline data (n = 17)**		**Follow-up data (n = 16)**		**Between-group changes**	
	**Exercise**	**No exercise**	**Exercise**	**No exercise**	**Difference**	**p-value**
**Gait outcomes**						
Peak KAM (%BW*ht)	3.75 (0.91)	3.38 (0.78)	3.70 (0.91)	3.21 (0.95)	0.04 (-0.64, 0.72)	0.91
KAM impulse (%BW*ht*sec)	1.13 (0.40)	1.32 (0.41)	1.01 (0.35)	1.24 (0.55)	–0.05 (–0.23, 0.32)	0.72
Walking speed (m/s)	1.18 (0.23)	1.03 (0.17)	1.28 (0.18)	1.04 (0.16)	0.08 (–0.02, 0.18)	0.11
**Strength outcomes**						
Knee extension torque (Nm/kg)	1.25 (0.34)	0.98 (0.25)	1.35 (0.39)	0.97 (0.23)	0.10 (–0.25, 0.06)	0.19
Knee flexion torque (Nm/kg)	1.06 (0.33)	0.82 (0.29)	1.12 (0.35)	0.70 (0.48)	0.18 (–0.41, 0.06)	0.12
Hip abduction torque (Nm/kg)	0.78 (0.19)	0.59 (0.26)	0.87 (0.28)	0.69 (0.13)	–0.02 (–0.14, 0.18)	0.77
						
**Urinary markers**						
uCTX-II (log ng/mmol creatinine)	5.40 (0.81)	5.97 (0.57)	5.32 (0.93)	6.25 (0.68)	–0.33 (–0.71, 0.04)	0.11
uC2C (log μg/mmol creatinine)	2.45 (0.68)	2.46 (0.76)	2.57 (0.81)	2.71 (0.58)	–0.10 (–0.35, 0.16)	0.73
**Serum markers**						
sHA (log U/L)	3.47 (0.93)	3.80 (0.96)	3.26 (1.13)	4.21 (0.86)	–0.79 (–1.67, 0.08)	0.10
sCOMP (log U/L)	2.20 (0.21)	2.26 (0.17)	2.11 (0.24)	2.36 (0.13)	–0.16 (–0.30,–0.02)	0.04
sCPII (log U/L)	6.56 (0.19)	6.44 (0.53)	6.50 (0.36)	6.71 (0.40)	–0.34 (–0.94, 0.24)	0.27
**Ratios**						
uCTX-II:sCPII	–1.16 (0.74)	–0.46 (0.49)	–1.18 (0.97)	–0.46 (0.81)	0.01 (–0.63, 0.66)	0.97
uC2C:sCPII	–4.11 (0.69)	–3.98 (1.19)	–3.93 (0.98)	–4.01 (0.73)	0.22 (–0.59, 1.03)	0.61
sHA:sCPII	–3.09 (0.99)	–2.64 (0.87)	–3.24 (1.37)	–2.50 (0.77)	–0.45 (–1.43, 0.53)	0.39

Group comparisons (exercise – no exercise) denote the difference in mean change (95% CIs). Between-group mean differences for gait and strength data are unadjusted, while biomarker data for each log-transformed biomarker and ratio using linear regression modeling while adjusting for age and sex. Note that negative log-transformed values indicate that the absolute ratio was less than 1.0, with greater negative values indicating a smaller ratio of the degradation biomarker to the synthesis biomarker sCPII. Thus, improvements in the ratio of degradation to synthesis would be reflected in smaller negative values at follow-up.

## Discussion

This pilot study provides new information regarding the characteristics of articular cartilage biomarkers relevant to the study and treatment of knee OA. Specifically, this study provides the first data detailing the relationship between multiple OA biomarkers and a measure of dynamic knee joint load – a potential mechanism of OA biomarker production – as well as changes in biomarker concentrations following exercise. Both study objectives taken together, these data provide some support to further explore the utility of uCTX-II and sCOMP as biomarkers relevant to knee OA.

Previous animal studies have shown a direct relationship between load magnitude and articular cartilage degradation [22]. The data from the present study provide some evidence that higher musculoskeletal loading can be associated with increased circulating levels of uCTX-II, though a causative relationship cannot be claimed based on the current data. Furthermore, recent evidence suggests that uCTX-II levels may arise from bone as well as cartilage [23]; however, higher loading applied to the bone would presumably increase uCTX-II levels under the same mechanism as uCTX-II derived from cartilage. Though the KAM is a well-accepted and valid measure of medial compartment joint load [10] with significant relationships with many clinical outcomes specific to knee OA in the medial compartment [8,24-29], it does not represent the total load within the knee joint [30] nor does it account for any potential changes in joint contact force that may result from increased muscle activity [31]. Further, though a significant relationship was observed between KAM impulse and uCTX-II at baseline, this relationship became non-significant when adjusting for disease severity and walking speed, and no relationship was found when examining the peak KAM value. Although dynamic knee joint load may play a role in the production of biomarkers – in particular, uCTX-II – it is not the only factor involved in this process and evaluation of a single component of overall joint load (KAM) does not necessarily represent the forces experienced by the cartilage in their entirety. It is clear that more research needs to be done to understand better the relationship between knee joint biomechanics and OA biomarkers.

The second objective of this study was to examine changes in biomarker concentrations following an exercise intervention. Results provide some evidence of a beneficial role of exercise on joint integrity at the cartilage level. Specifically, those in the exercise group demonstrated reduced mean sCOMP levels following the intervention compared to those in the control group. Previous studies have shown reductions in sCOMP following a single muscle strengthening session [32], though increases in sCOMP levels immediately following moderate exercise have also been reported [17]. Given that no significant between-group differences in muscle strength or KAM were observed, it is unlikely that the reductions in sCOMP were due to reductions in joint load. Indeed, baseline results from this study did not provide evidence of a relationship between dynamic joint load during walking and sCOMP levels. This is in contrast to uCTX-II and may suggest that production of uCTX-II and sCOMP occurs due to different mechanisms. This hypothesis cannot be tested using the current study design or data and requires further research.

Though the current results provide new information, this study does have some limitations. First, the small sample size in this pilot study may have reduced statistical power and the ability to make more definitive conclusions. Nevertheless, despite the small sample size, we did find some statistically significant results. Also, the biomarker concentrations measured in this study represent systemic levels that could theoretically have arisen from any number of joints in the body. However, the normal turnover of type II collagen in the body is relatively low, suggesting that significant changes in systemic levels may be expected to be due to pathological turnover from a single joint [33]. Finally, we chose a BMI cut-off of 35 kg/m^2^ to decrease skin movement artifact during walking, as is commonly used in motion analysis studies measuring the KAM [34-36] to maximize gait data accuracy. However, given that many people with knee OA are overweight or obese, the results of this study cannot necessarily be generalized to the entire knee OA population, and our findings must be viewed in light of this limitation.

## Conclusions

This study provides initial evidence of a potential relationship between loading in the knee joint during walking and circulating levels of biomarkers associated with articular cartilage degradation, specifically uCTX-II. A beneficial effect of strengthening exercises on cartilage health as evidenced by reduced levels of circulating sCOMP was also concluded from the results, though the mechanism of this finding is unknown. Further research with more subjects and a longer intervention period would provide verification of these findings and enhance our understanding of the utility of biomarkers in the diagnosis of knee OA as well as their potential as outcome measures following treatment.

## Competing interests

TS is a cofounder and minor shareholder in AnaMar. JC has received research grants from Centocor Research & Development Inc and from Amgen Inc. No other authors have declared a conflict of interest with this work.

## Authors’ contributions

MAH conceived the study and was assisted in study design by JC. CLP was the blinded assessor for all outcome measurements. TS, VBK, and JLH were involved in the analysis of samples for biomarker concentrations. SP was the unblinded research coordinator involved in recruitment and group allocation. ECS performed the statistical analysis. MAH drafted the manuscript while all other authors provided critical revision of the article for important intellectual content and gave final approval of the manuscript.

## Pre-publication history

The pre-publication history for this paper can be accessed here:

http://www.biomedcentral.com/1471-2474/14/115/prepub
